# Computational design of molecular motors as nanocircuits in Leishmaniasis

**DOI:** 10.12688/f1000research.10701.2

**Published:** 2017-08-03

**Authors:** Dipali Kosey, Shailza Singh

**Affiliations:** 1 National Centre for Cell Science, NCCS Complex, SP Pune University Campus, Pune, India

**Keywords:** Leishmaniasis, nanocircuit, synthetic biology, molecular motor

## Abstract

Cutaneous leishmaniasis is the most common form of leishmaniasis, caused by *Leishmania major *and is spread by the bite of a sandfly *.*This species infects the macrophages and dendritic cells Due to multi-drug resistance, there is a need for a new therapeutic technique. Recently, a novel molecular motor of *Leishmania*, Myosin XXI, was classified and characterized. In addition, the drug resistance in this organism has been linked with the overexpression of ABC transporters. Systems biology aims to study the simulation and modeling of natural biological systems whereas synthetic biology deals with building novel and artificial biological parts and devices  Together they have contributed enormously to drug discovery, vaccine design and development, infectious disease detection and diagnostics. Synthetic genetic regulatory networks with desired properties, like toggling and oscillation have been proposed to be useful for gene therapy. In this work, a nanocircuit with coupled bistable switch – repressilator  has been designed, simulated in the presence and absence of inducer, *in silico,* using Tinker Cell. When inducer is added, the circuit has been shown to produce reporter at high levels, which will impair the activity of Myosin XXI and ABC transporters. Validation of the circuit was also performed using GRENITS and BoolNet. The influence of inducer on the working of the circuit, i.e., the type of gene expression, response time delay, the steady states formed by the circuit and the quasipotential landscape of the circuit were performed. It was found that the addition of inducer reduced the response time delay in the graded type of gene expression and removed the multiple intermediate attractors of the circuit. Thus, the inducer increased the probability of the circuit to be present in the dominant stable state with high reporter concentration and hence the designed nanocircuit may be used for the treatment of leishmaniasis *.*

## Introduction

Leishmaniasis is a neglected tropical disease, caused by the protozoan parasite of the genus *Leishmania*. It mainly infects macrophages and dendritic cells of the immune system. Although several drugs are available for treatment, the rapid development of resistance in *Leishmania* to these drugs constantly demands new therapies ( Hadighi *et al.*, 2006). Of the three major types of Leishmaniasis viz (cutaneous, mucocutaneous and visceral leishmaniasis), cutaneous Leishmaniasis is caused by the species *Leishmania major*.

A novel molecular motor, Myosin XXI, has been identified in *Leishmania* and has been characterized recently. Its main role is suggested to be membrane anchorage and intracellular trafficking activity ( Katta *et al.*, 2010). Studies on Myosin XXI have shown that it is very important for the survival of the parasite and that it is the only myosin isoform present in the organism ( Batters *et al.*, 2014). It is also present only in the *Leishmania* genus, with less than 35% identity with other myosin isoforms. Its phosphorylation and dephosphorylation may be by Myosin Heavy Chain Kinase and Protein Phosphatase 2A (PP2A), respectively ( Batters *et al.*, 2014). Although the complete structure of Myosin XXI has not yet been reported, the various motifs present in it have been identified. Among these, there are six calmodulin binding (CB) motifs, which function by binding to calmodulin in the presence of calcium. This binding regulates Myosin XXI motility during various cellular functions. Amino acids 809–823 form the most potential CB motif. Therefore, antisense RNA to this CB motif will inhibit its translation and impair the motility of Myosin XXI. Also, the exposure to high PP2A levels will cause dephosphorylation of myosin, hindering its normal activity ( Batters *et al.*, 2014). Thus, the intracellular trafficking activity of Myosin XXI will be affected to a great extent, leading to the death of the parasite.

According to Katta *et al.* (2009) Myosin XXI is the only myosin isoform present in *Leishmania*. It is also exclusively a Leishmanial protein product. Attempts to obtain a Myosin XXI null mutant were unsuccessful ( Katta *et al.*, 2009), due to ploidy generation, indicating its essentiality for the organism. Further, the reduction of its levels has been found to result in a loss of endocytosis within the parasite’s flagellar pocket and impairment of other intracellular trafficking processes ( Katta *et al.*, 2009). Therefore, the specificity and essentiality of the target supports the selection of Myosin XXI as the target. In addition, though RNA silencing machinery is absent in *L. major*, antisense RNA has been used for interference with snoRNA in this species ( Liang *et al.*, 2005), showing that ssRNA silencing is possible in this species. Antisense RNA of the CB motif may directly impede the motility of the myosin XXI by interfering with the translation of the motif.

The resistance shown by *Leishmania* to the current therapeutic drugs is a rising concern among those combating the neglected disease, Leishmaniasis. As an approach to devise a novel therapeutic strategy and to fight leishmanial drug resistance, this study aims to target the molecular motor, Myosin XXI, as well as ABC transporters of *L. major*. The basic idea of this study is that impairments of the targets can be brought about by either antisense RNA for the most potential calmodulin binding motif (amino acids 809–823) of Myosin XXI or PP2A, which dephosphorylates Myosin XXI and ABC transporters.

Even though numerous studies have been performed on *L. major*, virtually no study is available on its molecular motor, Myosin XXI. Ever since Foth *et al*, 2006 established a new myosin class, Myosin XXI, for the previously unclassified trypanosomatid myosin, attempts have been made to understand it. Still in-depth knowledge of its characteristics is unknown, including its structure and mechanical properties. Exploring this novel molecular motor deeper might lead to newer therapeutic approaches for leishmaniasis, and to the best of our knowledge this is the first study on treatment of Leishmaniasis by targeting Myosin XXI. The use of a nanocircuit comprising a coupled bistable switch and repressilator for the treatment of disease is a novel idea, promising a whole new vista for therapeutic approaches.

## Methods

### Two strategies for the computational design of the nanocircuit

**Figure 1.**
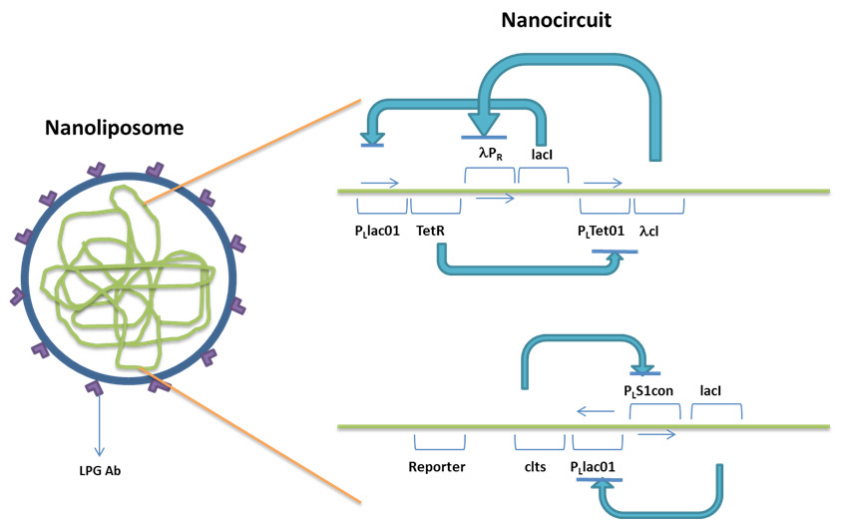
Nanocircuit encapsulated in an Ab coated nanoliposome.

A coupled bistable switch and repressilator, with the bistable switch under the control of repressilator can be delivered using a liposome (of nano size) with antibodies (Ab) targeting the lipophosphoglycan (LPG) displayed on an *L. major* infected macrophage. The liposome will be designed to withstand the acidic conditions of the phagolysosome of targeted macrophages. The circuit with bistable switch under the control of repressilator is given in Figure 1.

The repressilator has TetR (represses P _L_Tet01) under the control of the P _L_lac01 promoter, lacI (represses P _L_lac01) under the control of λP _R_, λcI (represses λP _R_), which is under the control of P _L_Tet01. All the repressilator proteins repress each other in a cyclic fashion. The bistable switch comprises of cIts (represses P _L_s1con), which is under the control of P _L_lac01 and lacI (represses P _L_lac01) under the control of P _L_s1con. P _L_lac01 is repressed by lacI of both the repressilator and bistable switch. The reporter gene is placed downstream of P _L_lac01.

The addition of an inducer of PLTet01, namely aTc, will increase the production of λcI, which will inhibit λP _R_. As production of lacI of the repressilator is stopped, some of the repression on P _L_lac01 will be released. cIts represses the production of lacI of the bistable switch. Thus, the reporter gene present downstream of P _L_lac01 will be expressed in increased levels.

The expression will be kept under control as the repression of lacI production in repressilator will release the repression on TetR (represses P _L_Tet01), which will counteract with the inducer aTc.

The choice of the reporter gene in the nanocircuit may be based on one of the following strategies.

**Figure 2.**
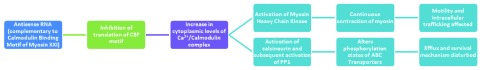
Flow chart of strategy I. CBF, Calmodulin Binding Motif; PP1, protein phosphatase 1; ABC transporters, ATP-binding cassette transporters.

***Strategy I ( Figure 2).*** The reporter gene may be devised such that its transcription results in ssRNA (antisense RNA), which will be complementary to the mRNA strand coding for the amino acids 809–823 – LQWVEEASNMFPDF ( Batters *et al.,* 2014). The inhibition of this calmodulin binding (CB) motif in Myosin XXI will result in the hindrance in CB, necessary for the motility of the parasite. There will be an increase in the levels of calmodulin calcium complex in the system. The calcium calmodulin complex activates the myosin heavy chain kinases, which will result in the continuous contraction of myosin. Thus, the motility and intracellular trafficking of the parasite will be disturbed. Also, the complex of calcium and calmodulin will choose to bind to other CB proteins, namely calcineurin. Activation of calcineurin will in turn activate PP1. PP1 and PP2A function as molecular switch that reciprocally regulate the eukaryotic phosphatases, hence suggesting the fact that PP1 modulates PP2A activity in cells. ( Ohki *et al.*, 2001). As more PP1 is activated, phosphorylation of ABC transporter proteins necessary for its regulation will also be affected, thus disturbing the efflux of molecules. Consequently, *L. major* will be put under stress as the motility, efflux of endogenous metabolites; survival mechanisms and intracellular trafficking are affected, leading to death of the parasite.

**Figure 3.**
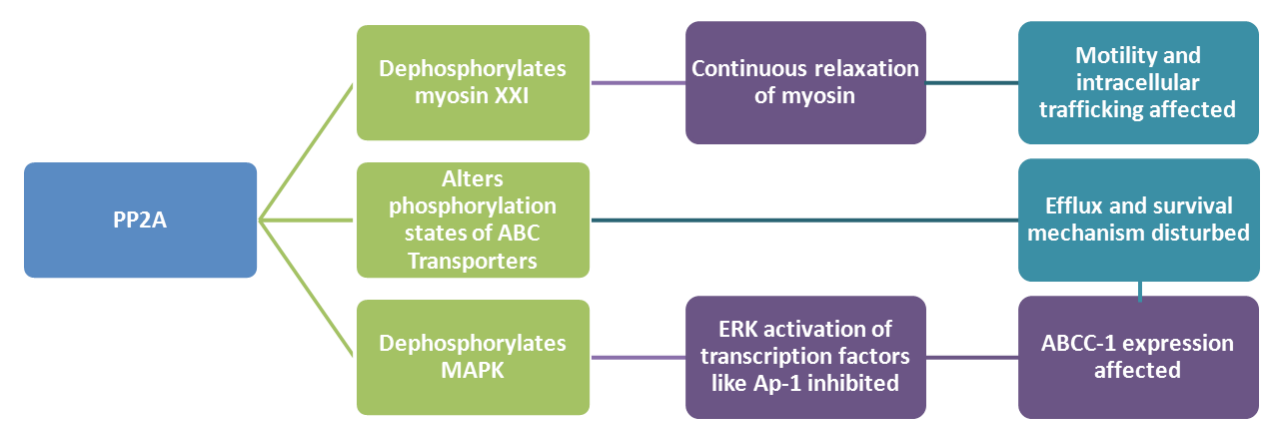
Flow chart of strategy II. MAPK, Mitogen activated protein kinase; *ABCC1,* ATP Binding Cassette Subfamily C Member 1; PP2A, Protein phosphatase-2A; Ap-1, Activator protein 1; ABC transporters, ATP-binding cassette transporters

***Strategy II ( Figure 3).*** The reporter gene can be devised to synthesize Protein Phosphatase 2A (PP2A), which dephosphorylates the myosin heavy chain. Excess production of PP2A by the circuit will result in continuous relaxation of myosin. This will in turn affect the parasite’s motility and intracellular trafficking, endangering its survival. PP2A will also disturb the phosphorylation states needed for ABC transporter activity ( Berridge MJ, 2010). Also, dephosphorylation of mitogen-activated protein kinase (MAPK), by PP2A, has been noted in several studies ( Toivola & Eriksson, 1999). Several studies have reported that since extracellular signaling kinase (ERK) is involved in activation of transcription factors, like *Ap-1*, which is associated with *ABCC1* (multidrug resistance protein-1) expression, dephosphorylation by PP2A may also have an effect on ABC transporter expression ( El Azreq *et al.*, 2012; Xu *et al.*, 2006). Thus, the efflux of molecules will be affected. The trafficking mechanism of *L. major*, its survival mechanisms and drug resistance property breaks down as a result. Thus, *L. major* will cease to exist, unable to cope up with the build-up of stress.

**Figure 4.**
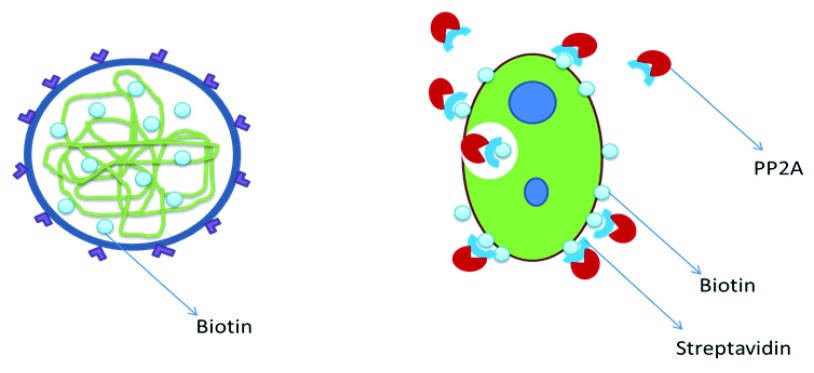
Use of Biotin-streptavidin for internalization of reporter.

***Cofactor – streptavidin and biotin.*** Streptavidin has been selected as a cofactor to ensure the internalization of the reporter produced by the circuit. This is based on the fact that biotin-streptavidin-based isolation of *Leishmania* has been widely practiced. Biotin, if encapsulated inside the nanoliposome containing the nanocircuit, will become attached to the surface proteins of *L. major* inside the macrophages. Therefore, the streptavidin-reporter complexes attaches with this bound biotin, finally leading to internalization of complex ( Figure 4).

### Designing and simulation of the nanocircuit using Tinker Cell

The nanocircuit was designed using different biological parts available in Tinker Cell ( http://www.tinkercell.com/). The transcriptional repression processes were also indicated in the circuit. Sequences for the different parts of the gene modules were obtained from various sources: the reporter DNA and protein sequences were obtained from NCBI; the DNA and protein sequences for repressor proteins, ribosome binding sites, terminators were obtained from Registry for Standard Biological Parts; and the promoter sequences with binding strengths for the repressor proteins were acquired using TRANSFAC and TFBind ( Supplementary File 1). These sequences were entered in appropriate places in the Text Attributes dialog box in Tinker Cell. Steady state parameter scan was performed for the designed circuit. Simulation of the working of the circuit was done by altering the values of protein degradation rates, rbs and promoter strengths, dissociation constants, and hill coefficients, until the desired graph was obtained. Inducer was added to PLTet01 of the circuit, and the simulation was performed again. The final results were exported in SBML format for further analysis. The SBML files were validated using an online SBML validator ( http://sbml.org/Facilities/Validator/).

### Validation of the nanocircuit using Genetic Regulatory Network Inference using Time Series (GRENITS) and BoolNet

The SBML files of the simulated circuit (with and without inducer) were loaded in COPASI (v4.18; software for simulation and modeling of biochemical networks; http://copasi.org/) and time course data for a period of 10s was generated for the protein concentrations. The acquired files were analyzed by GRENITS package (v1.24.0; https://www.bioconductor.org/packages/release/bioc/html/GRENITS.html) for the circuit’s convergence and network link probabilities. Inferred network of the circuit components was obtained from the network links with probability greater than 0.8. The files were also analyzed by BoolNet (v2.1.3; https://cran.r-project.org/web/packages/BoolNet/index.html). The possible attractor states formed by the circuit, the probability of Boolean network transitions and network wiring were found. Robustness of the circuit for around perturbations was also checked. Attractor results from BoolNet were exported as .net format and the circular layout of the state transitions were obtained using Pajek (v4.10; http://mrvar.fdv.uni-lj.si/pajek/).

### Studies on circuit behavior using Berkeley Madonna

The SBML files of the designed and simulated circuit (with and without inducer) were loaded in COPASI, and the ODE files of the circuit were exported in .mmd format. The ODE files were loaded to Berkeley Madonna (v8.3.18; www.berkeleymadonna.com). The Bistable switch - Repressilator coupling equation was included as follows:

                cod1 = LcI1+ (PLlac01_strength*rs4_c*rs5_c * DefaultCompartment)

The ODEs were integrated using Euler’s method from 0 to 10s. A plot of Antisense RNA or PP2A *vs* time was obtained. The plots for the circuit with and without inducer were compared to determine the type of gene expression in the nanocircuit and to understand the effect of inducer on the response time delay. A plot of Antisense RNA or PP2A *vs* LacI3, showing the phase plane of the two proteins, was obtained.

A nullcline was plotted using the option Nc in Berkeley Madonna. Another nullcline was obtained by plotting several trajectories from different initial conditions using the option Ic.

The point of intersection of the two nullclines shows the steady state of the designed circuit.

1. Difference equation for quasipotential, Vq = -(((Antisense_RNA_or_PP2A)^2) + ((lacI3)^2))*DT was included and integration was performed again.
2. A plot of antisense RNA or PP2A *vs* LacI3 vsVq was obtained.
3. The values were exported from Berkeley Madonna and 3D plots (contour plot, 3D mesh, linearized 3D mesh) of the quasipotential landscape were obtained using SigmaPlot 12.0 ( http://www.sigmaplot.co.uk/). The landscapes for the circuit with and without inducer were compared.

Source codes (zipped file) The file contains all the source codes and other files for the nanocircuit designing, simulation and validation. Click here for additional data file. Copyright: © 2017 Kosey D and Singh S 2017 Data associated with the article are available under the terms of the Creative Commons Zero "No rights reserved" data waiver (CC0 1.0 Public domain dedication).

## Results

### Design and simulation of the nanocircuit

The circuit was designed using Tinker Cell, a CAD software. All the sequences for the different parts of the gene modules were entered as detailed in the Methods section.

**Figure 5.**
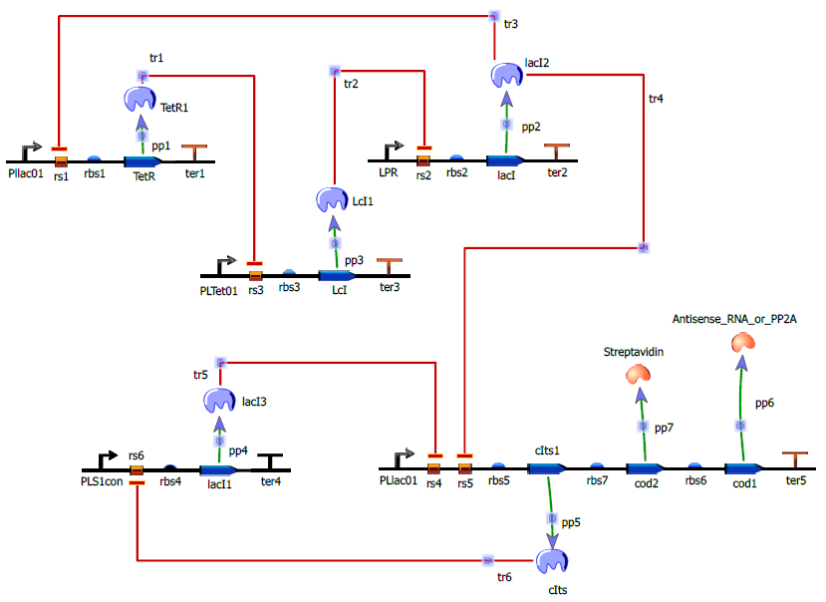
Design of nanocircuit.

Figure 5 shows the designed nanocircuit without inducer.

The upper portion of the circuit forms the repressilator with TetR, Lambda cI, LacI protein repressing each other in a cyclic fashion. The lower portion forms the Bistable switch comprised by cIts and lacI mutually repressing each other. LacI of repressilator represses PLLac01 of Bistable switch forming the coupling between repressilator and Bistable switch. The reporters Antisense RNA or PP2A, as well as streptavidin, are placed downstream of P _L_Lac01 of Bistable switch.

Simulation was performed and the desired graph was obtained for the values shown in Table 1.

**Figure 6.**
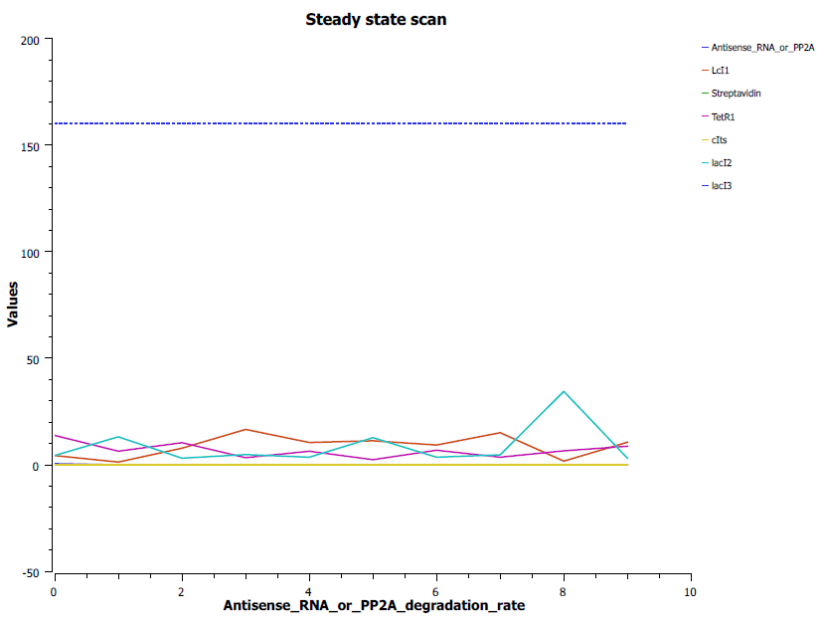
Simulation of working of the nanocircuit.

Figure 6 shows the working of the circuit in the absence of the inducer aTc. cIts and so Antisense RNA or PP2A (and streptavidin) are in off stage, due to the repression by LacI2, while LacI3 is on. The repressilator proteins TetR, LcI, LacI2 are forming oscillations.

**Table 1.** Values of parameters for the nanocircuit.

Parameter	Value
Antisense or PP2A degradation rate	0.1
LPR strength	4.222
LcI1 degradation rate	0.1177
PLS1con strength	4.99948
PLTet01 strength	4.8866
PLlac01 strength	4
Pllac01 strength	5.0688
Streptavidin degradation rate	0.1
TetR1 degradation rate	0.0954
cIts degradation rate	0.1099
lacI2 degradation rate	0.1093
lacI3 degradation rate	0.093
rbs1_strength	1.00011
rbs2_strength	1.10002
bs3_strength	0.85
rbs4_strength	0.596
rbs5_strength	0.60001
rbs6_strength	0.6
rbs7_strength	0.59648
tr1_kd	1
tr1_h	2
tr2_kd	1
tr2_h	2
tr3_kd	1.216
tr3_h	2.54
tr4_kd	1
tr4_h	2
tr5_kd	1
tr5_h	2
tr6_kd	1.234
tr6_h	2

**Figure 7.**
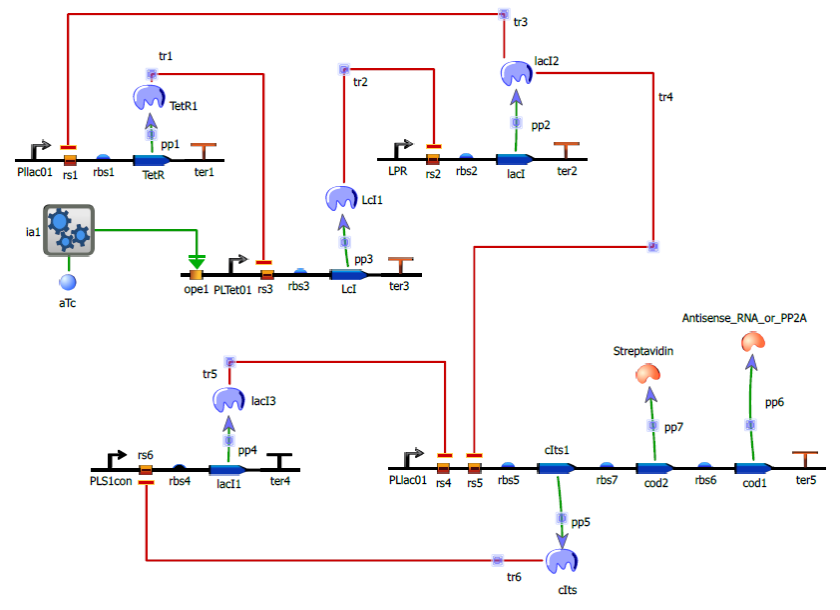
Design of circuit with inducer.

Figure 7 shows the circuit with inducer aTc added, which will reduce the repression caused by TetR on P _L_Tet01.

Table 2 shows the values of the three parameters of the induced activation, for which the desired graph was obtained. The rest of the values of the circuit were kept constant. The graph ( Figure 7) shows the working of the circuit after the addition of aTc.

**Table 2.** Values of parameters of induced activation for nanocircuit with inducer.

Parameter	Value
ia1_kd	0.0007
ia1_rep_kd	1.12063
ia1_rep_h	1.96

**Figure 8.**
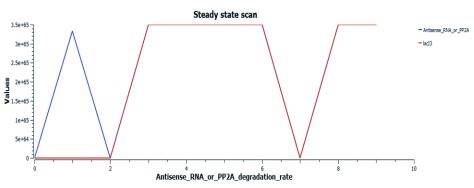
Simulation of working of the nanocircuit after addition of inducer.

The reporters (Antisense RNA or PP2A) are switched on when Antisense RNA or PP2A degradation rate is in the range of 0–2s, while the lacI3 gets switched off, as in Figure 8.

**Figure 9.**
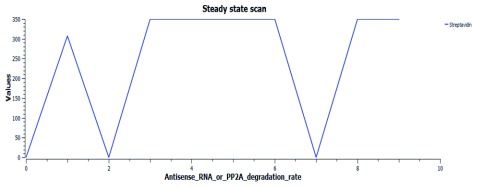
Behavior of Streptavidin after addition of inducer.

The cofactor Streptavidin also peaks in the same range ( Figure 9).

**Figure 10.**
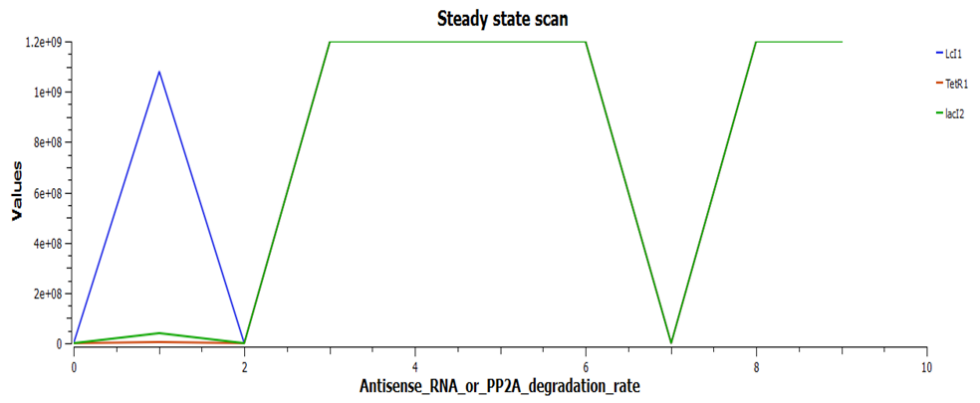
Oscillation of repressilator proteins after addition of inducer.

The repressilator proteins TetR, LcI and LacI2 form limit cycle oscillations in this range ( Figure 10).

Thus, the circuit was designed and the working in the absence and presence of inducer. The corresponding values of the parameters were also noted.

### Validation of the nanocircuit

The validation of the circuit with and without inducer was performed using GRENITS and BoolNet package in R (v3.2.2; https://cran.r-project.org/).

**(i)    Convergence**

Convergence of the circuit was checked using Monte Carlo Markov Chain Algorithm by GRENITS. Various parameters, such as indicator variables of Gibbs sampler, coefficient of regression, network connectivity parameter, precision of each regression and intercepts of regression of Chain 1 and Chain 2 of state transitions were analyzed.

**Figure 11.**
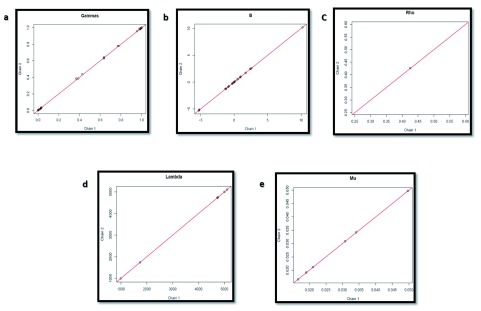
Convergence plots of the nanocircuit without inducer.

The graphs in Figure 11 shows the convergence of indicator variables of Gibbs sampler (Gamma), coefficients of regression (B), network connectivity parameter (Rho), precision of each regression (Lambda) and intercepts of each regression (Mu) of state transition chain 1 and 2, thus validating the circuit without inducer.

**Figure 12.**
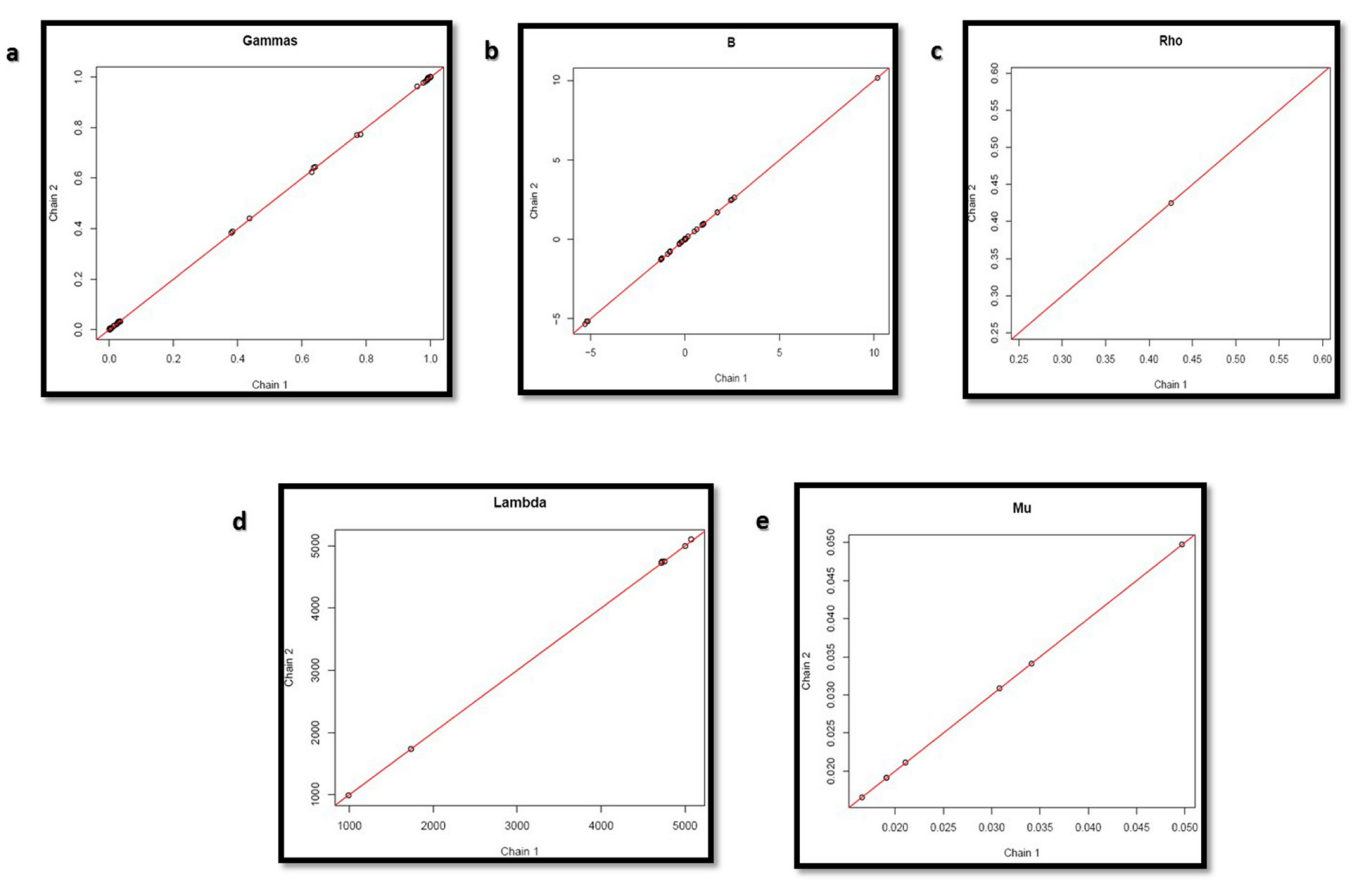
Convergence plots of the nanocircuit after addition of inducer.

Graphs in Figure 12 shows that convergence of the parameters was also obtained for the circuit with inducer, thus validating it.

**(ii)    Network link analysis plots**

The links between the different genetic components in the circuit were analyzed using GRENITS package. Links with probability > 0.8 were considered to infer the network.

**Figure 13.**
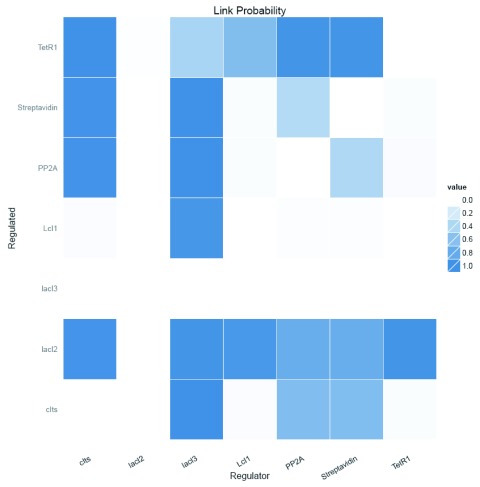
Link probabilities of the nanocircuit.

Figure 13 shows the link probabilities between the components. From the plot, it is evident that LacI3 of the bistable switch is the most important and strong regulator of the circuit, as it regulates the expression of all other proteins.

**Figure 14.**
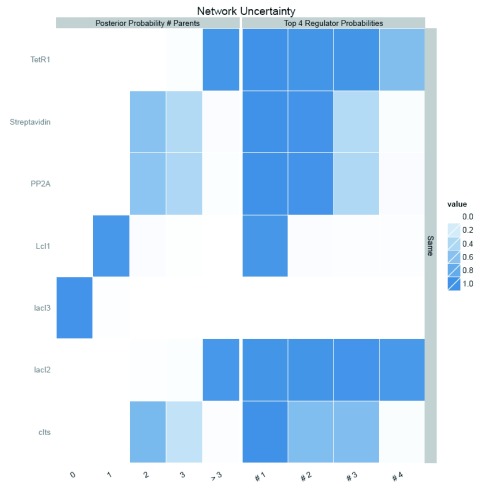
Network uncertainty of the nanocircuit.

Figure 14 shows the number of regulating parents for each of the genes and the probabilities of each of the parent regulating it. It shows that LacI3 has no parent regulating it.

**Figure 15.**
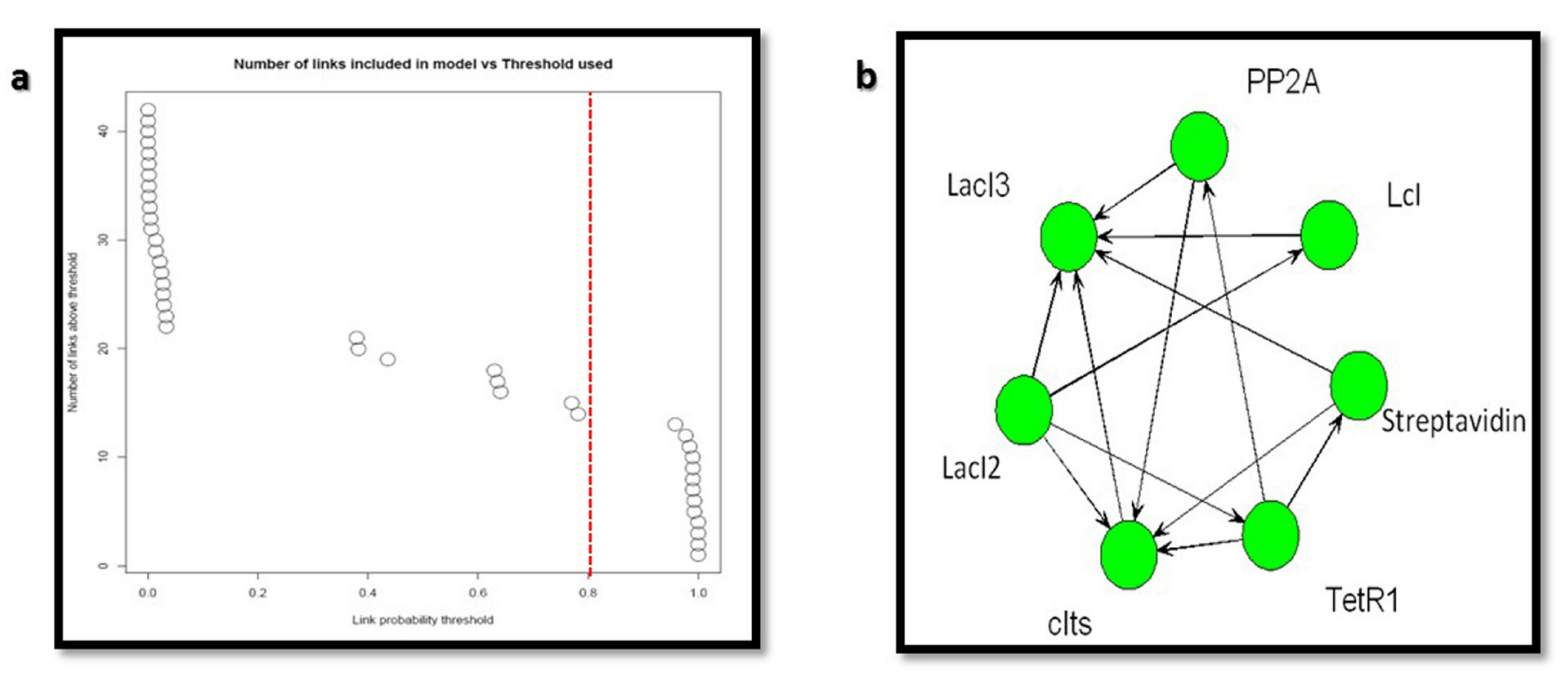
( **A**) Link probability threshold and ( **B**) the inferred network for the nanocircuit.

The inferred network between the genetic components considering the links with probabilities > 0.8 ( Figure 15A) is shown in Figure 15B

**Figure 16.**
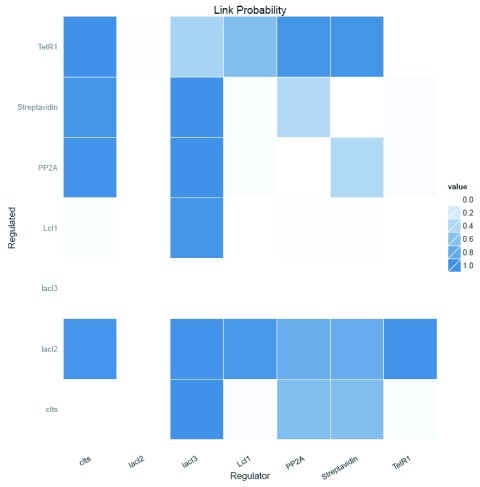
Link probabilities of the nanocircuit with inducer.

**Figure 17.**
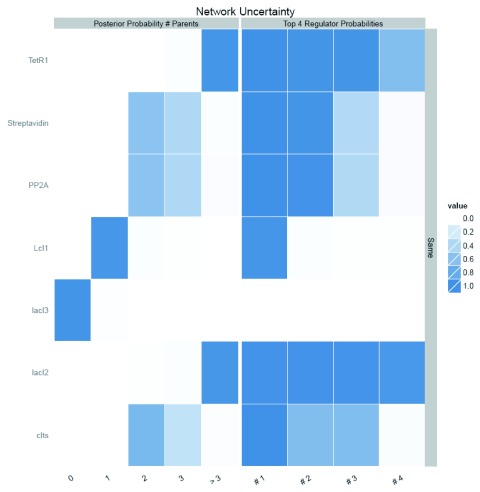
Network uncertainty of the nanocircuit with inducer.

Similarly, links in the nanocircuit with inducer were analyzed using GRENITS, which resulted in the same results; LacI3 was the main regulator ( Figure 16) and had no parents regulating it ( Figure 17).

**Figure 18.**
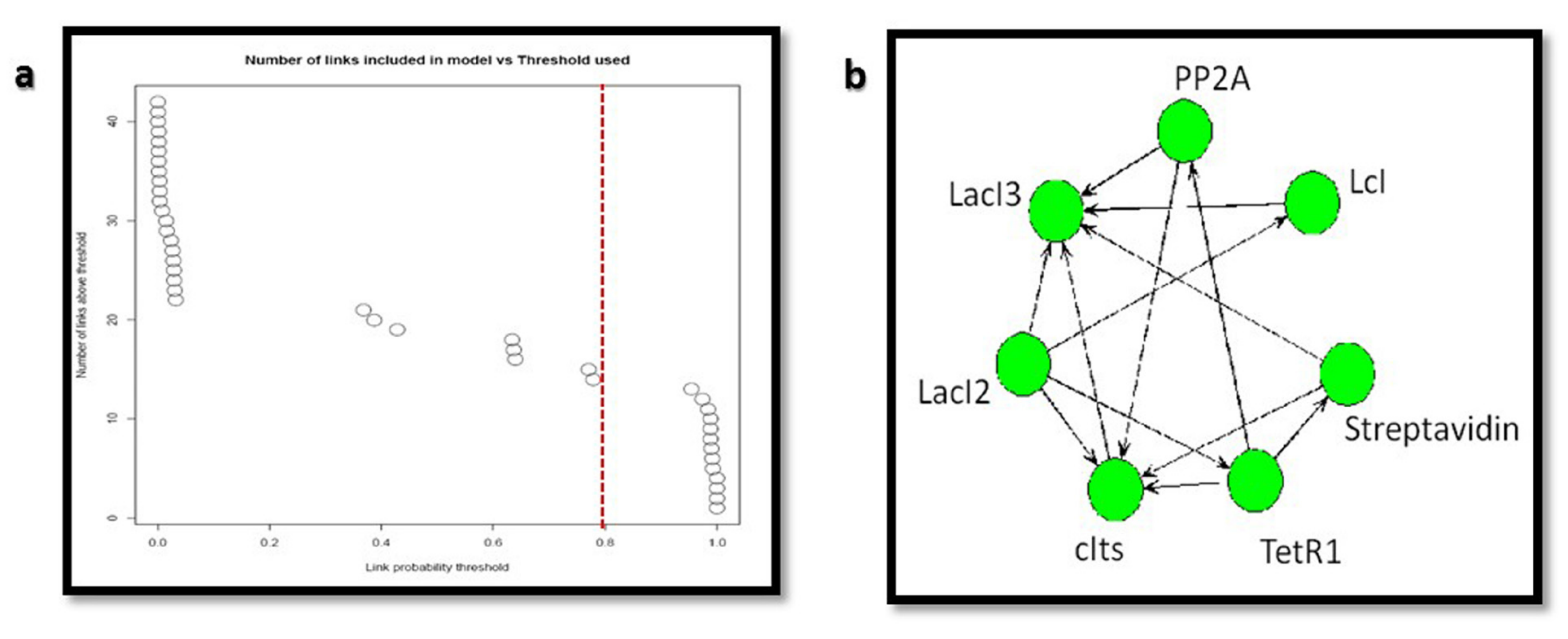
( **A**) Link probability threshold and ( **B**) the inferred network for the nanocircuit with inducer.

The inferred network from the links with probability > 0.8 ( Figure 18A) is shown in Figure 18B.

The inferred network shows that all the components are linked to one another with high probability. This proves the non-randomness of the network.

**(iii)    Attractor state analysis**

The possible attractor states formed by the circuit were determined using BoolNet to visualize the switching on and off of the genes comprising the circuit.

**Figure 19.**
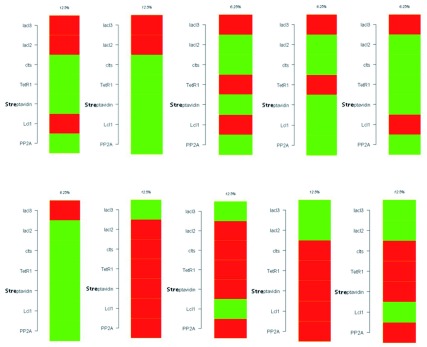
Attractor states of the nanocircuit.

Figure 19 shows the ten possible attractor states with continuous switching of the repressilator genes between on and off states. The reporters are off when LacI3 is on and they are on when LacI3 is off, which is an evidence for the toggling between the genes of the bistable switch.

**Figure 20.**
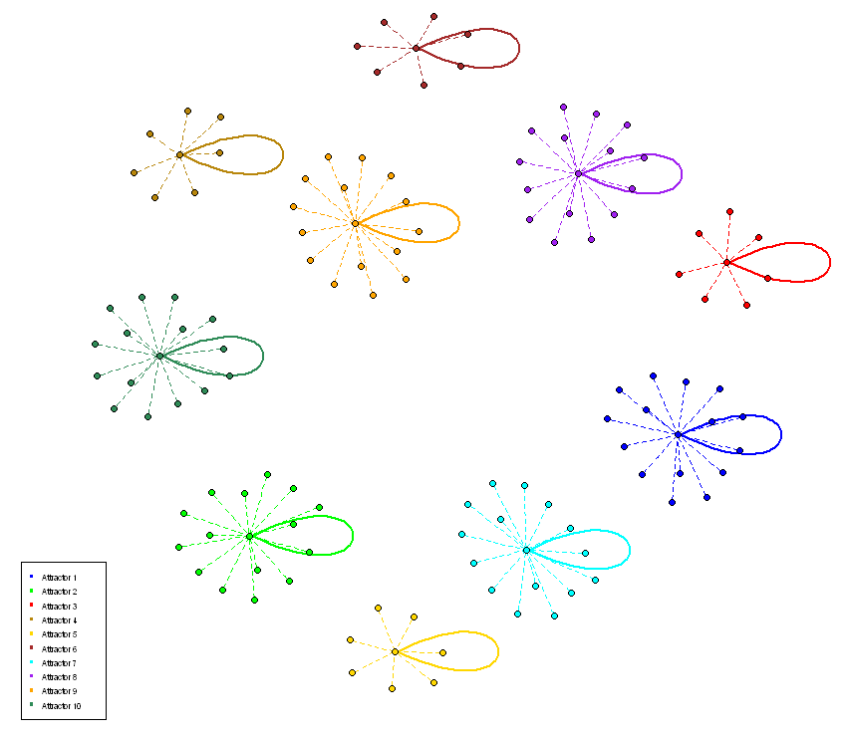
State graph of the nanocircuit.

Figure 20 depicts the ten attractors with either 8 or 16 states forming the basin of attractor.

**Figure 21.**
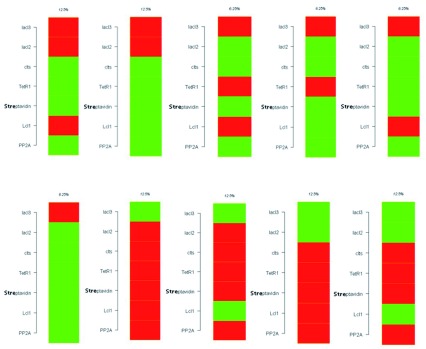
Attractor states of the nanocircuit with inducer.

**Figure 22.**
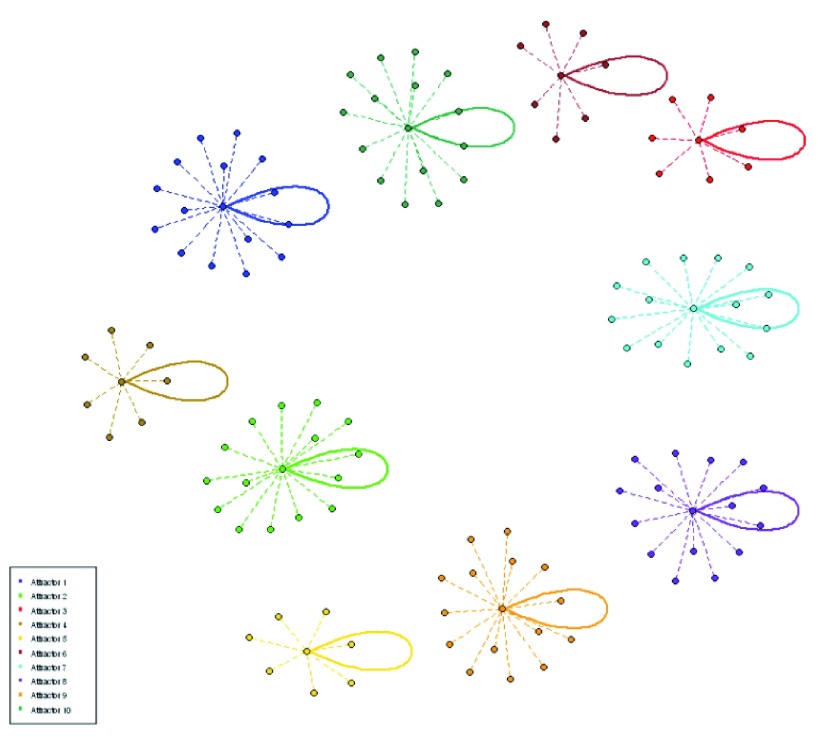
State graph of the nanocircuit with inducer.

The attractor state analysis of the nanocircuit with inducer also yielded 10 attractors with 8 or 16 states forming the attractor basin ( Figure 21 and Figure 22).

**Figure 23.**
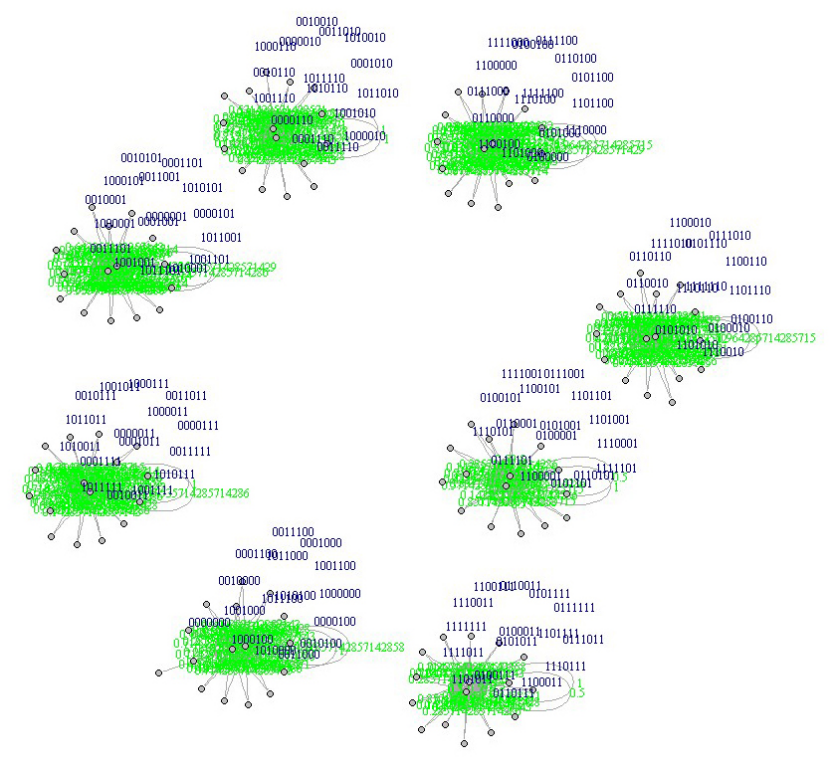
Probability Boolean Transitions.

Figure 23 illustrates the Probability Boolean Network transitions in the circuit with and without inducer. It gives the binary representation of the attractors and its basins.

**(iv)    Robustness analysis**

The robustness of the circuit to 10 different perturbations was checked using BoolNet (a state transition table was generated, one or several transitions are perturbed randomly and the gene transition function are robust from the modified transition table).

**Figure 24.**
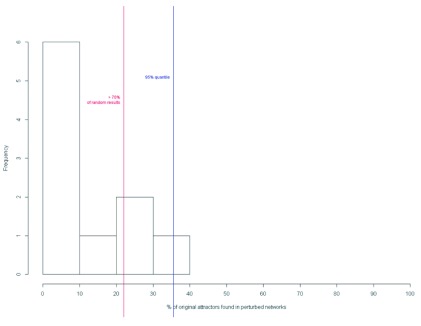
Percentage of original attractors in perturbed networks (nanocircuit).

Figure 24 shows the percentage of attractors present in the perturbed networks of the circuit: 10% of original attractors were present in 6 perturbed networks; 20% in 1; 30% in 2; and 40% in 1 network. Hence, the result showed that 70% of the random results had the original attractors undisturbed.

**Figure 25.**
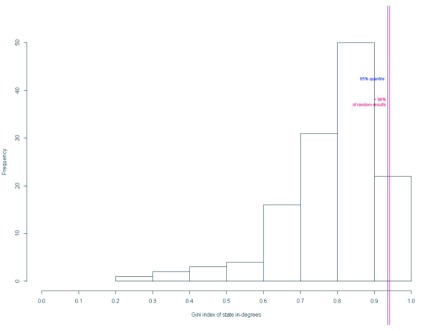
Gini index of state-in degrees (nanocircuit).

Figure 25 shows the Gini index of state-in degrees of the circuit. The state-in degrees of a particular state is the number of transitions leading to it. The Gini index of the in-degrees is returned as a characteristic value of the network. It is a measure of inequality. If all states have an in-degree of 1, the Gini index is 0. If all state transitions lead to one single state, the Gini index is 1 ( Kim *et al.,* 2007). The higher the Gini index, the higher the number of transitions leading to it. This parameter is measured based on the fact that in biological networks, many state transitions lead to the same states. Figure 25 show that 96% of the states have a high Gini index, proving that the designed circuit is a biologically valid network and not a random network.

**Figure 26.**
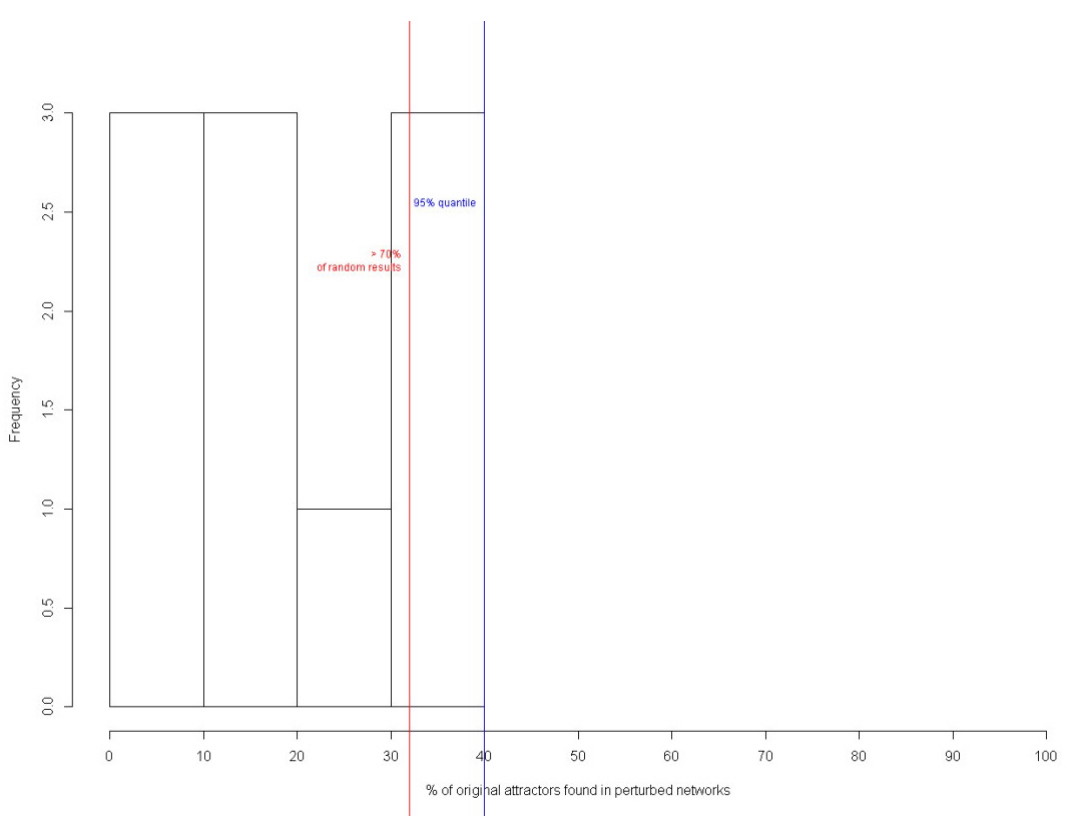
Percentage of original attractors in perturbed networks (nanocircuit with inducer).

**Figure 27.**
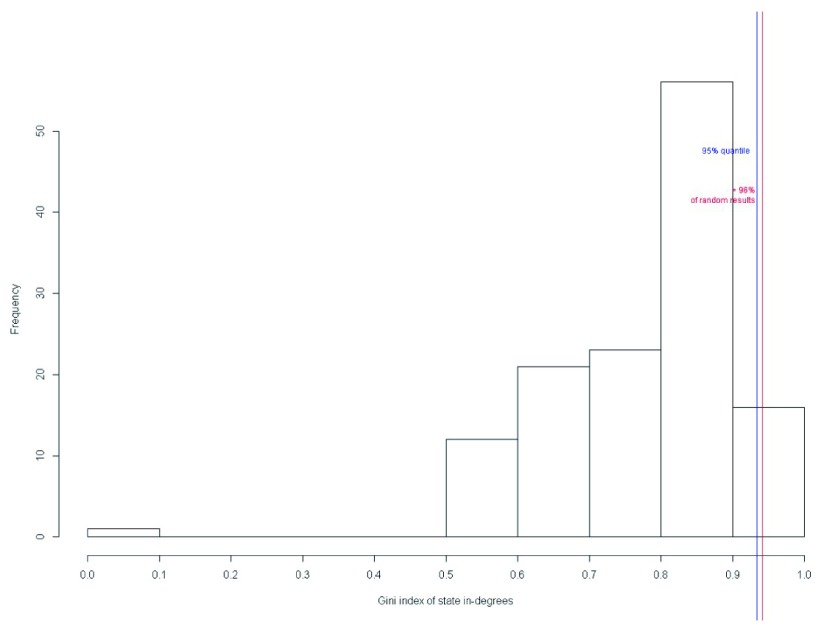
Gini index of state-in degrees (nanocircuit with inducer).

Similar results were obtained for nanocircuit with inducer with BoolNet. In total, 70% of the perturbed networks had the original attractors ( Figure 26), while 96% of the attractors had high Gini index of state-in degrees ( Figure 27). Hence, the biological validity of the circuit has been proved.

**(v)    Network wiring**

**Figure 28.**
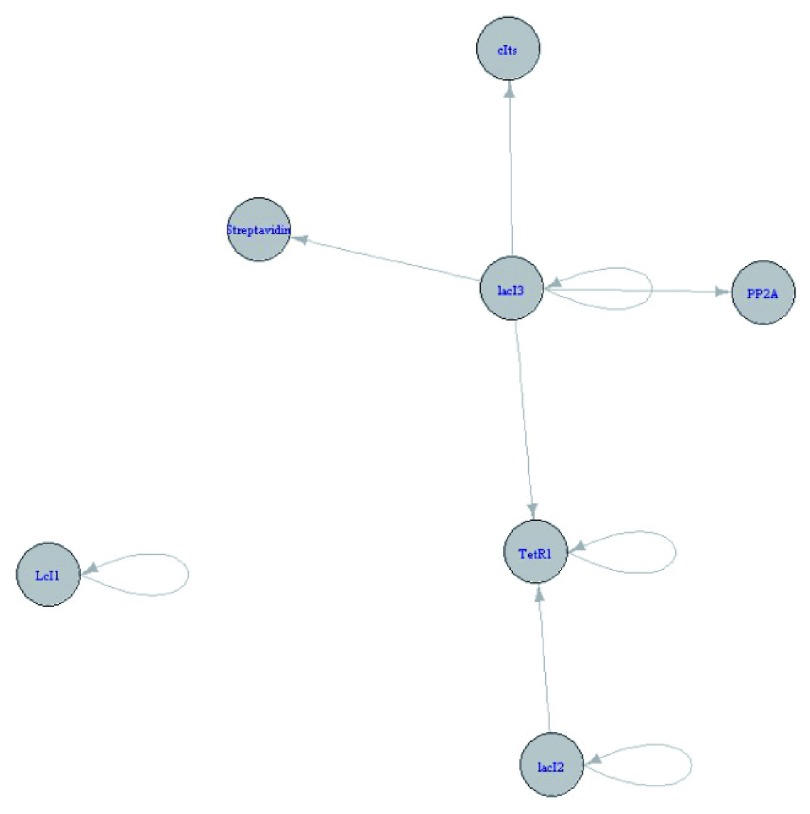
Network wiring in the nanocircuit.

Based on the transition function of each gene found from the links between the gentic components, network wiring was made using BoolNet. From the network ( Figure 28), it is evident that LacI3 controls the expression of other proteins of the circuit and is also a function of itself, while not being controlled by other components.

**(vi)    Circular layout of the state transitions**

**Figure 29.**
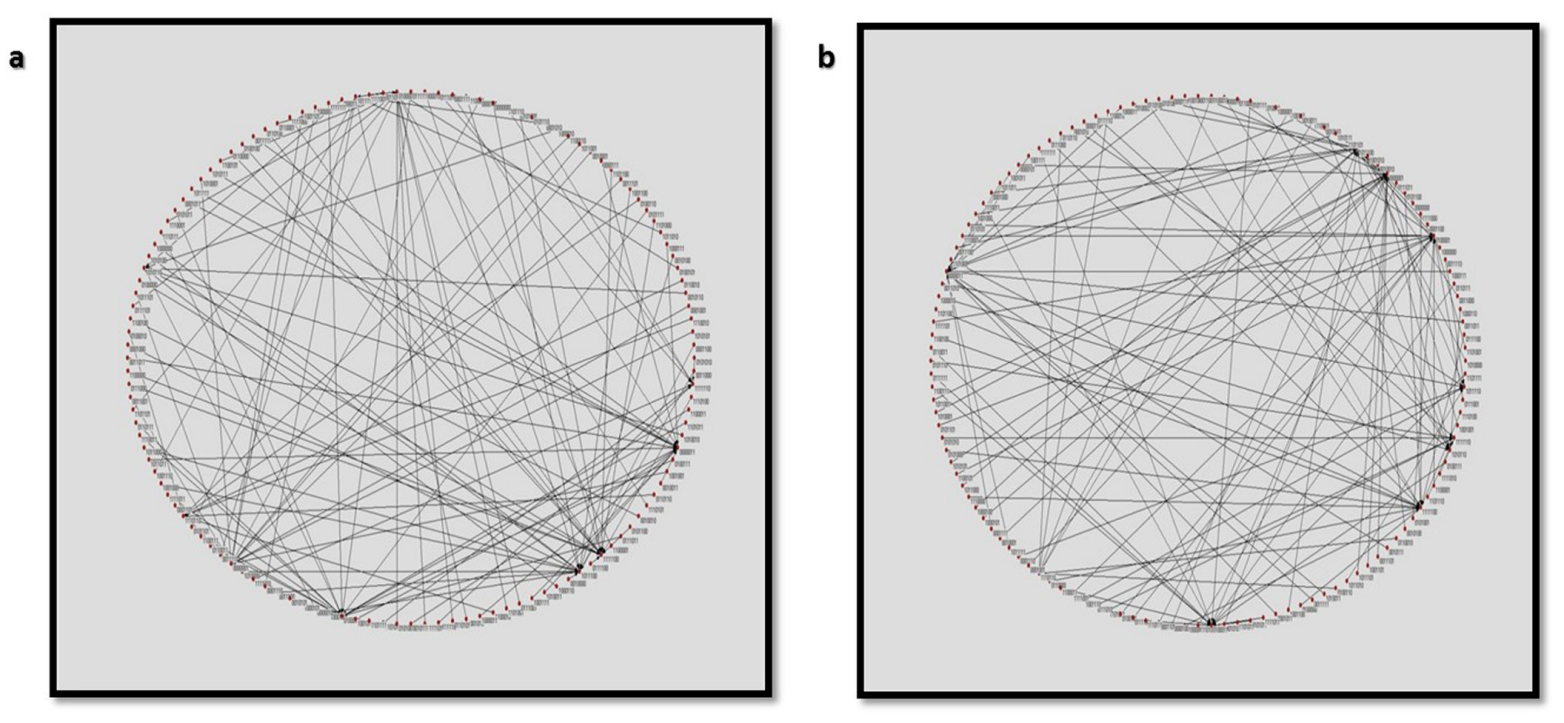
Circular layout of the state transitions of the nanocircuit ( **A**) without and ( **B**) with inducer.

Figure 29A and B shows the circuit layout of the 2 ^129^ transitions and the probable attractors in the circuit and the circuit with inducer, respectively.

Thus, several other relationships are possible that can lead to a difference in network behaviour. Here the concept of modularity is of paramount importance. Modularity is the degree to which a system’s components can be segregated or recombined. In synthetic biology, each module has a fixed arrangement pertaining to which it exhibits a characteristic behaviour; if this modular arrangement is altered there are perturbations in the global dynamics, oscillations and kinetics of a system. A second round of simulation performed without parameter scan sketched 16 attractor states which portrayed a stochastic behaviour of attractor probabilities. The state matrix which we framed from the graph of transition states made it is pretty much clear that both proteins decay over the same time period in our analysis when the circuit is initially inactive (no stimuli) and then switch to the active state. This behavioural features work in a contrast fashion when the system switches in an opposite direction i.e., from initially active to inactive state. Our Boolean analysis solidified the idea that below a threshold value, expression is never induced. The next set of quantitative analysis which was performed using the GRENITS module gave results which were congruent with the qualitative justifications.

### Studies on circuit behavior

**(i)    Gene expression and response time delay**

**Figure 30.**
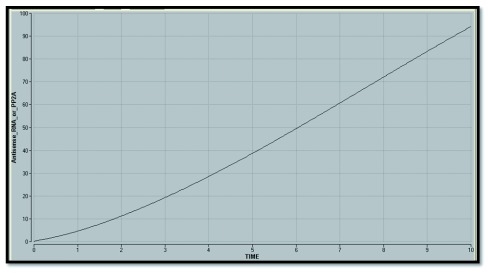
Response time delay in nanocircuit.

**Figure 31.**
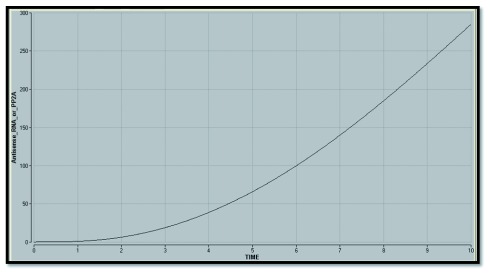
Response time delay in nanocircuit with inducer.

The plot of Antisense RNA or PP2A *vs* time ( Figure 30 and Figure 31) was obtained using Berkeley Madonna. The gradual increase in the concentration of the reporter shows that there is graded expression in the circuit. Comparison between the two graphs shows that in addition to a high level production of reporter, the response time delay has also been reduced by the addition of inducer.

### Determination of steady state

**Figure 32.**
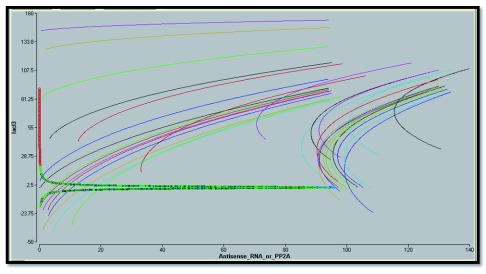
Geometric nullclines of the nanocircuit.

**Figure 33.**
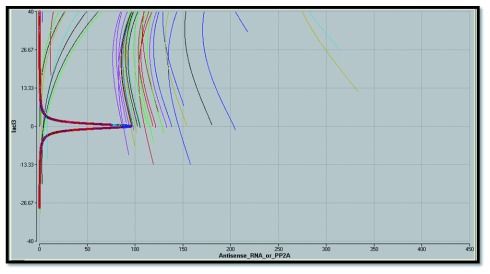
Geometric nullclines of the circuit with inducer.

Considering the biological fact that at equilibrium protein concentration does not change over time, nullclines (dx/dt = 0 or dy/dt = 0) have been plotted for reporters and LacI3 in the phase plane of the two proteins, i.e. d(Antisense RNA or PP2A)/dt = 0 or d(LacI3)/dt=0). The point of intersection indicates the steady states (d(Antisense RNA or PP2A)/dt = 0 and d(LacI3)/dt=0)). The nullclines of a bistable switch genes intersect thrice with two stable steady states. From Figure 32 and Figure 33, it is evident that the designed nanocircuit favors only one steady state with high reporter concentration and low LacI3 concentration. The individual trajectories in the graph show the evolution of the system from their respective initial conditions.

From the sharp threshold of steady state in equilibrium conditions, it is apparent that the circuit is ultrasensitive.

**(ii)    Quasipotential landscape**

To evaluate the dynamics and evaluation of the circuit under non-equilibrium conditions, quasipotential landscape was mapped. It also gives a quantitative measure of the epigenetic landscape. The directionality of the circuit can also be studied.

**Figure 34.**
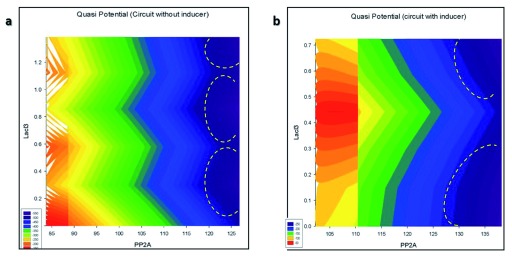
Contour plot of the Quasipotential of nanocircuit ( **A**) without and ( **B**) with inducer.

Figure 34 shows the contour plot of quasipotential energy at different levels of reporter and LacI3. The regions marked by yellow lines are local minima in the quasipotential surface. It corresponds to the stable states as the areas of minimum potential that have the highest probability of occupancy. This plot also shows that the circuit works by autoactivation. Autoactivation of the reporter leads to amplification of transient differences in expression between the reporter and LacI3.

**Figure 35.**
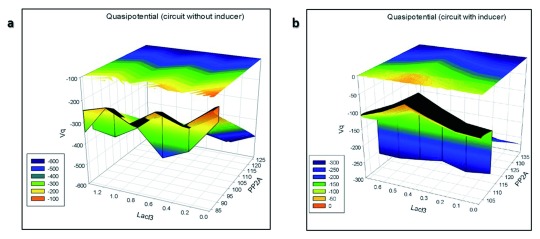
3D Quasipotential landscape of the nanocircuit ( **A**) without and ( **B**) with inducer.

**Figure 36.**
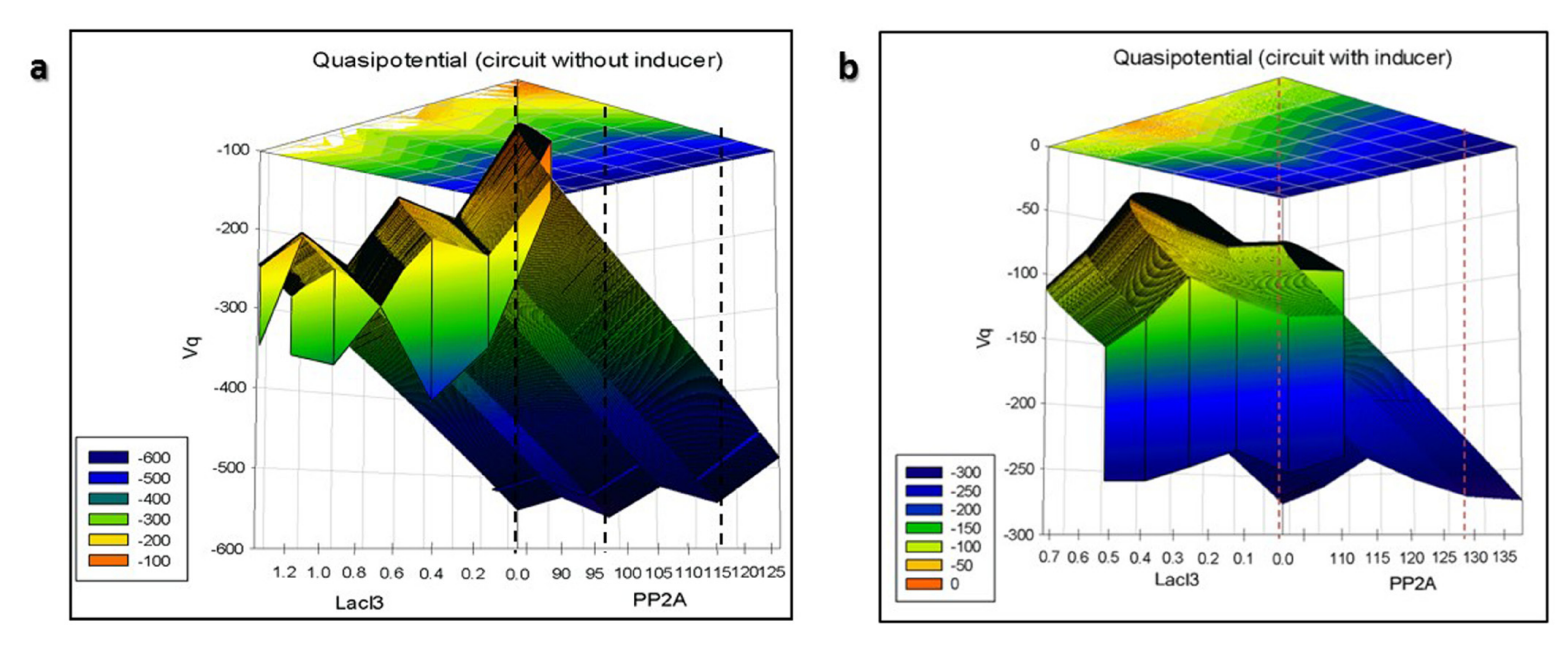
Linearized quasipotential landscape of the nanocircuit ( **A**) without and ( **B**) with inducer.

The 3D quasipotential landscape is given in Figure 35. The branching valleys and ridges depict the stable cellular states and the barriers between those states, respectively. The “deeper” valleys in the figure are associated with a higher probability of occupancy than the “shallower” valleys. Figure 35 shows that autoactivation has caused multistability in the circuit. A circuit with an initial condition of low reporter and high LacI3 will flow down-hill to the intermediate stable state with minimal quasi potential ( Figure 36). For the circuit to flow to the stable steady state from the intermediate state with high reporter and low LacI3, it has to cross the potential barrier between the two states, for which additional reactions are required which will need energy. Therefore, the probability of this transition is less. In Figure 36, it can be seen that there is a smooth down-hill flow to the stable steady state, with high reporter levels and low LacI3 levels from all initial conditions when inducer is added. There is translational burst of the reporter, leading it to the dominant attractor. Therefore, the probability of occupancy of this stable steady state is very high. The number of intermediate stable states with minimal potential has been reduced by the inducer.

Figure 36 shows the linearized 3D plot from which the relative distances between the stable states can be inferred.

In the absence of inducer, there is a higher number of intermediate attractors closely spaced ( Figure 36A). When inducer is added, the dominant attractor is placed well away from the initial condition with a smooth down-hill flow ( Figure 36B). This occurs due to the high level expression of the reporter placed downstream of the active promoter and low levels of regulatory LacI3, which is upstream of this promoter.

Thus, it can be concluded that the circuit works by autoactivation of the reporter, while repressing LacI3. This has led to multistability. Addition of inducer reduces the multistability caused by the autoactivation in the circuit and smooths the undulating quasipotential landscape. Therefore, the circuit will occupy the stable state of high reporter levels with high probability.

## Discussion

The basic idea of this study is that impairments of the targets can be brought about by either antisense RNA for the most potential calmodulin binding motif (amino acids 809-823) of Myosin XXI or protein phosphatase 2A (PP2A; dephosphorylates Myosin XXI and ABC transporters). The nanocircuit behaves as a nanomachine when a chemical gradient, e.g. inducer, is given and produces high levels of reporter. Antisense RNA of the CB motif may directly impede the motility of the Myosin XXI by interfering with the translation of the motif. It may also affect the phosphorylation levels of myosin by increasing the cytoplasmic levels of Ca2+/calmodulin complex, in turn activating the myosin heavy chain kinase that phosphorylates myosin. Abnormal phosphorylation of Myosin XXI, due to high levels of ssRNA made by the circuit, leads to continuous contraction which hampers its activity. Similarly, high levels of PP2A made available by the nanocircuit may cause continuous relaxation by dephosphorylating myosin XXI and inhibit phosphorylation by Myosin Heavy Chain Kinase.

In nutshell, the simulation, validation and behavior studies of the nanocircuit confirmed the hypothesis that the designed nanocircuit is a robust biological network and, when inducer is added, will lead to high amounts of the reporter with reduced response time delay. While the circuit by itself favors only this stable steady state, though with multiple attractors, the inducer increases the probability of occupancy of this state under various initial conditions, through a translational burst of the reporter. The intracellular trafficking, motility by the myosin XXI and the metabolite efflux, survival mechanism of the ABC transporters will be hindered, leading to the death of the leishmanial parasite. Future studies will involve *in vitro* and *in vivo* validation of the circuit. The nanocircuit would be constructed in a plasmid and delivered using Lipophosphoglycan-antibody coated nanoliposomes. The method adopted helps to devise a nanocircuit for treating Leishmaniasis which may behave as a nanomachine.

## Data availability

The data referenced by this article are under copyright with the following copyright statement: Copyright: © 2017 Kosey D and Singh S

Data associated with the article are available under the terms of the Creative Commons Zero "No rights reserved" data waiver (CC0 1.0 Public domain dedication). 

**Dataset 1: Source codes (zipped file).** The file contains all the source codes and other files for the nanocircuit designing, simulation and validation. doi, 10.5256/f1000research.10701.d150383 ( Kosey & Singh, 2017).

## Supplementary material

**Supplementary File 1: Source sequences.** All the sequences for the different parts of the gene modules are available.
