# Supplementary material for: Computational design of molecular motors as nanocircuits in Leishmaniasis
Source: F1000Res. 2017 Aug 3;6:94. Originally published 2017 Jan 31. [Version 2] doi: 10.12688/f1000research.10701.2 (PMC5580435; doi:10.12688/f1000research.10701.2)
Supplement: Supplementary file 2 [file f1000research-6-13332-s0001.tgz › 1e0e4bce-6404-4d8d-9572-cc72a204789b.docx]

1. **Sequences of Reporter Proteins**
2. **ssRNA**

**Camodulin Binding motif -** LQWVEEASNMFPDF

**mRNA –** CUGCAGUGGGUGGAAGAAGCGAGCAACAUGUUUCCGGAUUUU

**DNA strand coding coding for mRNA** –

CTGCAGTGGGTGGAAGAAGCGAGCAACATGTTTCCGGATTTT

**DNA strand coding for Antisense RNA** –

TTTTAGGCCTTTGTACAACGAGCGAAGAAGGTGGGTGACGTC

1. **PP2A**

>gi|157874292|ref|XM_001685578.1| Leishmania major strain Friedlin putative protein phosphatase 2A catalytic subunit (LMJF_32_3040) mRNA, complete cds

ATGTTAACAGAAATATTGGACATTGACGAGCACCTCGCCACCCTCAACCGGTGCGAGAACATCGGCGAGG

CGGATGTGAAGCGGCTGTGCTTGAAGGCAAAGGAGATTTTCACGAGTGAGGAGAACGTGCACAAGATCCC

GGCTCCGTGCACCATCGTGGGCGACATCCATGGCCAATTCTACGACCTCCTGGAGCTCTTCCGCGTCGGT

GGCGAAATCCCGGACACAAACTACGTCTTCATGGGCGATTTCGTAGACCGAGGCTACCATAGCGTAGAGT

CGTTCCTGTTGCTACTTGTGCTCAAGATCAAGTACTCTCGTCGCGTTGCGCTGGTGCGCGGCAACCACGA

GTCGCGCCAAATTACGCAGGTGTACGGCTTCTACGATGAGTGCTTGCGCAAGTACGGCAGCATCAACGTG

TGGCGCTACTGCACGGACGTGTTCGATCTCTTGCCCCTAGCGAGCGTCGTGGAAGGGAAGATCTTCTGCG

TGCACGCTGGTCTGTCGCCATCGATACAGACGGTGGACCAGATGCGTTCCATCCGCCGTAACTGCGAGGT

GCCGCACGAAGGCGCCATGTGCGACTTGCTCTGGTCCGATCCGGAGGACATTGACGGCTGGGGTCTGTCG

CCGCGCGGCGCCGGCTACCTGTTCGGCGGTGATGTCGTGTGCCAGTTCAACGAGACAAACAAGCTAGACC

TCATCTGCCGCTCCCATCAGCTGGTGATGGAGGGATACAAGGCTATGTTCAATGACACGCTTGTGACGGT

CTGGTCGGCGCCCAACTACTGTTACCGCTGTGGTAACGTTGCGTCTATTTTGGAGCTGGACGAGCACCTG

AACAAGAACTTCAAGATTTTTGAGGCTGCGCCGGCAGAGGCGCGCGAGGCTGGTCAGCGGAACGAGGTAC

CAGCCTACTTCCTTTAG

>gi|157874293|ref|XP_001685630.1| putative protein phosphatase 2A catalytic subunit [Leishmania major strain Friedlin]

MLTEILDIDEHLATLNRCENIGEADVKRLCLKAKEIFTSEENVHKIPAPCTIVGDIHGQFYDLLELFRVGGEIPDTNYVFMG

DFVDRGYHSVESFLLLLVLKIKYSRRVALVRGNHESRQITQVYGFYDECLRKYGSINVWRYCTDVFDLLPLASVVEGKIFCV

HAGLSPSIQTVDQMRSIRRNCEVPHEGAMCDLLWSDPEDIDGWGLSPRGAGYLFGGDVVCQFNETNKLDLICRSHQL

VMEGYKAMFNDTLVTVWSAPNYCYRCGNVASILELDEHLNKNFKIFEAAPAEAREAGQRNEVPAYFL

1. **Sequences of Repressor Proteins**

**(i) TetR**

>gi|445048304:2257-2907 Escherichia coli 3.4880 gec34880.contig.122_1, whole genome shotgun sequence

TCAATCGTCACCCTTTCTCGGTCCTTCAACGTTCCTGACAACGAGCCTCCTTTTCGCCAATCCATCGACA

ATCACCGCGAGTCCCTGCTCGAACGCTGCGTCCGGACCGGCTTCGTCGAAGGCGTCTATCGCGGCCCGCA

ACAGCGGCGAGAGCGGAGCCTGTTCAACGGTGCCGCCGCGCTCGCCGGCATCGCTGTCGCCGGCCTGCTC

CTCAAGCACGGCCCCAACAGTGAAGTAGCTGATTGTCATCAGCGCATTGACGGCGTCCCCGGCCGAAAAA

CCCGCCTCGCAGAGGAAGCGAAGCTGCGCGTCGGCCGTTTCCATCTGCGGTGCGCCCGGTCGCGTGCCGG

CATGGATGCGCGCGCCATCGCGGTAGGCGAGCAGCGCCTGCCTGAAGCTGCGGGCATTCCCGATCAGAAA

TGAGCGCCAGTCGTCGTCGGCTCTCGGCACCGAATGCGTATGATTCTCCGCCAGCATGGCTTCGGCCAGT

GCGTCGAGCAGCGCCCGCTTGTTCCTGAAGTGCCAGTAAAGCGCCGGCTGCTGAACCCCCAACCGTTCCG

CCAGTTTGCGTGTCGTCAGACCGTCTACGCCGACCTCGTTCAACAGGTCCAGGGCGGCACGGATCACTGT

ATTCGGCTGCAACTTTGTCAT

>gi|256367701|ref|YP_003108192.1| TetR [Escherichia coli]

MFISDKVSSMTKLQPNTVIRAALDLLNEVGVDGLTTRKLAERLGVQQPALYWHFRNKRALLDALAEAMLAENHTHSVP

RADDDWRSFLIGNARSFRQALLAYRDGARIHAGTRPGAPQMETADAQLRFLCEAGFSAGDAVNALMTISYFTVGAVL

EEQAGDSDAGERGGTVEQAPLSPLLRAAIDAFDEAGPDAAFEQGLAVIVDGLAKRRLVVRNVEGPRKGDD

**(ii) LacI**

>BBa_K091121 Part-only sequence (1083 bp)

ATGAAACCAGTAACGTTATACGATGTCGCAGAGTATGCCGGTGTCTCTTATCAGACCGTTTCCCGCGTGGTGAACC

AGGCCAGCCACGTTTCTGCGAAAACGCGGGAAAAAGTGGAAGCGGCGATGGCGGAGCTGAATTACATTCCCAAC

CGCGTGGCACAACAACTGGCGGGCAAACAGTCGTTGCTGATTGGCGTTGCCACCTCCAGTCTGGCCCTGCACGCG

CCGTCGCAAATTGTCGCGGCGATTAAATCTCGCGCCGATCAACTGGGTGCCAGCGTGGTGGTGTCGATGGTAGAA

CGAAGCGGCGTCGAAGCCTGTAAAGCGGCGGTGCACAATCTTCTCGCGCAACGCGTCAGTGGGCTGATCATTAAC

TATCCGCTGGATGACCAGGATGCCATTGCTGTGGAAGCTGCCTGCACTAATGTTCCGGCGTTATTTCTTGATGTCTC

TGACCAGACACCCATCAACAGTATTATTTTCTCCCATGAAGACGGTACGCGACTGGGCGTGGAGCATCTGGTCGCA

TTGGGTCACCAGCAAATCGCGCTGTTAGCGGGCCCATTAAGTTCTGTCTCGGCGCGTCTGCGTCTGGCTGGCTGGC

ATAAATATCTCACTCGCAATCAAATTCAGCCGATAGCGGAACGGGAAGGCGACTGGAGTGCCATGTCCGGTTTTC

AACAAACCATGCAAATGCTGAATGAGGGCATCGTTCCCACTGCGATGCTGGTTGCCAACGATCAGATGGCGCTGG

GCGCAATGCGCGCCATTACCGAGTCCGGGCTGCGCGTTGGTGCGGATATCTCGGTAGTGGGATACGACGATACCG

AAGACAGCTCATGTTATATCCCGCCGTTAACCACCATCAAACAGGATTTTCGCCTGCTGGGGCAAACCAGCGTGGA

CCGCTTGCTGCAACTCTCTCAGGGCCAGGCGGTGAAGGGCAATCAGCTGTTGCCCGTCTCACTGGTGAAAAGAAA

AACCACCCTGGCGCCCAATACGCAAACCGCCTCTCCCCGCGCGTTGGCCGATTCATTAATGCAGCTGGCACGACAG

GTTTCCCGACTGGAAAGCGGGCAGTGA

>gi|251784242|ref|YP_002998546.1| LacI transcriptional repressor [Escherichia coli BL21(DE3)]

MKPVTLYDVAEYAGVSYQTVSRVVNQASHVSAKTREKVEAAMAELNYIPNRVAQQLAGKQSLLIGVATSSLALHAPSQI

VAAIKSRADQLGASVVVSMVERSGVEACKAAVHNLLAQRVSGLIINYPLDDQDAIAVEAACTNVPALFLDVSDQTPINSI

IFSHEDGTRLGVEHLVALGHQQIALLAGPLSSVSARLRLAGWHKYLTRNQIQPIAEREGDWSAMSGFQQTMQMLNEG

IVPTAMLVANDQMALGAMRAITESGLRVGADISVVGYDDTEDSSCYIPPLTTIKQDFRLLGQTSVDRLLQLSQGQAVKG

NQLLPVSLVKRKTTLAPNTQTASPRALADSLMQLARQVSRLESGQ

**(iii) Lambda cl**

>BBA_I741110 Part-only sequence (714 bp)

ATGAGCACAAAAAAGAAACCATTAACACAAGAGCAGCTTGAGGACGCACGTCGCCTTAAAGCAATTTATGAAAAA

AAGAAAAATGAACTTGGCTTATCCCAGGAATCTGTCGCAGACAAGATGGGGATGGGGCAGTCAGGCGTTGGTGC

TTTATTTAATGGCATCAATGCATTAAATGCTTATAACGCCGCATTGCTTGCAAAAATTCTCAAAGTTAGCGTTGAAG

AATTTAGCCCTTCAATCGCCAGAGAAATCTACGAGATGTATGAAGCGGTTAGTATGCAGCCGTCACTTAGAAGTGA

GTATGAGTACCCTGTTTTTTCTCATGTTCAGGCAGGGATGTTCTCACCTGAGCTTAGAACCTTTACCAAAGGTGATG

CGGAGAGATGGGTAAGCACAACCAAAAAAGCCAGTGATTCTGCATTCTGGCTTGAGGTTGAAGGTAATTCCATGA

CCGCACCAACAGGCTCCAAGCCAAGCTTTCCTGACGGAATGTTAATTCTCGTTGACCCTGAGCAGGCTGTTGAGCC

AGGTGATTTCTGCATAGCCAGACTTGGGGGTGATGAGTTTACCTTCAAGAAACTGATCAGGGATAGCGGTCAGGT

GTTTTTACAACCACTAAACCCACAGTACCCAATGATCCCATGCAATGAGAGTTGTTCCGTTGTGGGGAAAGTTATC

GCTAGTCAGTGGCCTGAAGAGACGTTTGGCTGA

>gi|425330686|ref|ZP_18718559.1| repressor protein CI [Escherichia coli EC1846]

MVQNEKVRKEFAQRLAQACKEAGLDEHGRGMAIARALSLSSKGVSKWFNAESLPRQEKMNALAKFLNVDVVWLQH

GTSLNGANDEDTLSFVGKLKKGLVRVVGEAILGVDGAIEMTEERDGWLKIYSDDPDAFGLRVKGDSMWPRIKSGEYVLI

EPNTKVFPGDEVFVRTVEGHNMIKVLGYDRDGEYQFTSINQDHRPITLPYHQVAKVEYVAGILKQSRHLDDIEAREWLK

SS

**(iv) cIts**

>BBa_K098997 Part-only sequence (714 bp)

ATGAGCACAAAAAAGAAACCATTAACACAAGAGCAGCTTGAGGACGCACGTCGCCTTAAAGCAATTTATGAAAAA

AAGAAAAATGAACTTGGCTTATCCCAGGAATCTGTCGCAGACAAGATGGGGATGGGGCAGTCAGGCGTTGGTGC

TTTATTTAATGGCATCAATGCATTAAATGCTTATAACGCCGCATTGCTTGCAAAAATTCTCAAAGTTAGCGTTGAAG

AATTTAGCCCTTCAATCGCCAGAGAAATCTACGAGATGTATGAAGCGGTTAGTATGCAGCCGTCACTTAGAAGTGA

GTATGAGTACCCTGTTTTTTCTCATGTTCAGGCAGGGATGTTCTCACCTGAGCTTAGAACCTTTACCAAAGGTGATG

CGGAGAGATGGGTAAGCACAACCAAAAAAGCCAGTGATTCTGCATTCTGGCTTGAGGTTGAAGGTAATTCCATGA

CCGCACCAACAGGCTCCAAGCCAAGCTTTCCTGACGGAATGTTAATTCTCGTTGACCCTGAGCAGGCTGTTGAGCC

AGGTGATTTCTGCATAGCCAGACTTGGGGGTGATGAGTTTACCTTCAAGAAACTGATCAGGGATAGCGGTCAGGT

GTTTTTACAACCACTAAACCCACAGTACCCAATGATCCCATGCAATGAGAGTTGTTCCGTTGTGGGGAAAGTTATC

GCTAGTCAGTGGCCTGAAGAGACGTTTGGCTGA

>gi|31790200|gb|AAP57959.1| temperature-sensitive repressor protein cIts [Mycobacterium phage L1cIts391]

MSGKIQHKAVVPAPSRIPLTLSEIEDLRRKGFNQTEIAELYGVTRQAVSWHKKTYGGRLTTRQIVQQNWPWDTRKPHD

KSKAFQRLRDHGEYMRVGSFRTMSEDKKKRLLSWWKMLRDDDLVLEFDPSIELYEGMAGGGFRYVPRGIEDDDLLIRV

NEHTNLTAEGELLWSWPDDIEELLSEP

1. **Sequences of Strong Promoters**
2. **TetR**

Acc ID: M00055 V$NMYC_01

Binding Strength - 0.8832122

Consensus Sequence: (+) NNNCACGTGNNN

Signal Sequence: TCGCGCGTGCAA

Promoter Strength - 0.143212

1. **LacI**

Acc ID :M00055 V$NMYC_01

Binding Strength - 0.8832122

Consensus Sequence: (+) NNNCACGTGNNN

Signal Sequences: TCGCGCGTGCAA

Promoter Strength - 0.143212

1. **Lambda cl**

Acc ID : M00174 V$AP1_Q6

Binding Strength - 0.9394512

Consensus Sequence : (-) NNTGACTCANN

Signal Sequence : AGTTAGTCATT

Promoter Strength - 0.179451

1. **cIts**

Acc ID : M00174 V$AP1_Q6

Binding Strength - 0.9394512

Consensus Sequence : (-) NNTGACTCANN

Signal Sequence : AGTTAGTCATT

Promoter Strength -0.179451

1. **Sequences of Ribosome Binding Sites (RBS)**

**RBS.1 (strong) – modified from R.Weiss**

BBa_B0030 (15 bp) Binding efficiency - 0.6

ATTAAAGAGGAGAAA

1. **Sequence of Terminator**

BBa_B0010 (80 bp)

CCAGGCATCAAATAAAACGAAAGGCTCAGTCGAAAGACTGGGCCTTTCGTTTTATCTGTTGTTTGTCGGTGAACGCTCTC

1. **Sequence of Cofactor**

Streptavidin

>gi|46740|emb|X03591.1| Streptomyces avidinii gene for streptavidin

CCCTCCGTCCCCGCCGGGCAACAACTAGGGAGTATTTTTCGTGTCTCACATGCGCAAGATCGTCGTTGCAGCCATC

GCCGTTTCCCTGACCACGGTCTCGATTACGGCCAGCGCTTCGGCAGACCCCTCCAAGGACTCGAAGGCCCAGGTCT

CGGCCGCCGAGGCCGGCATCACCGGCACCTGGTACAACCAGCTCGGCTCGACCTTCATCGTGACCGCGGGCGCCG

ACGGCGCCCTGACCGGAACCTACGAGTCGGCCGTCGGCAACGCCGAGAGCCGCTACGTCCTGACCGGTCGTTACG

ACAGCGCCCCGGCCACCGACGGCAGCGGCACCGCCCTCGGTTGGACGGTGGCCTGGAAGAATAACTACCGCAAC

GCCCACTCCGCGACCACGTGGAGCGGCCAGTACGTCGGCGGCGCCGAGGCGAGGATCAACACCCAGTGGCTGCT

GACCTCCGGCACCACCGAGGCCAACGCCTGGAAGTCCACGCTGGTCGGCCACGACACCTTCACCAAGGTGAAGCC

GTCCGCCGCCTCCATCGACGCGGCGAAGAAGGCCGGCGTCAACAACGGCAACCCGCTCGACGCCGTTCAGCAGTA

GTCGCGTCCCGGCACCGGCGGGTGCCGGGACCTCGGCC

>gi|14606|emb|CAA00084.1| streptavidin [Streptomyces avidinii]

MDPSKDSKAQVSAAEAGITGTWYNQLGSTFIVTAGADGALTGTYESAVGNAESRYVLTGRYDSAPATDGSGTALGWT

VAWKNNYRNAHSATTWSGQYVGGAEARINTQWLLTSGTTEANAWKSTLVGHDTFTKVKPSAASIDAAKKAGVNNG

NPLDAVQQ
