# Supplementary material for: Computational design of molecular motors as nanocircuits in Leishmaniasis
Source: F1000Res. 2017 Aug 3;6:94. Originally published 2017 Jan 31. [Version 2] doi: 10.12688/f1000research.10701.2 (PMC5580435; doi:10.12688/f1000research.10701.2)
Supplement: Peer review history

# Peer review: Referee response for version 2

**DOI:** 10.5256/f1000research.13332.r24789

**Published:** 2017-08-21

**Authors:** Jaime J. Seguel

**Affiliations:** 1 Department of Computer Science and Engineering, College of Engineering, University of Puerto Rico at Mayagüez, Mayagüez, Puerto Rico

I recommend indexing of the reviewed version of this article.

I have read this submission. I believe that I have an appropriate level of expertise to confirm that it is of an acceptable scientific standard.

# Peer review: Referee response for version 1

**DOI:** 10.5256/f1000research.11538.r23063

**Published:** 2017-07-31

**Authors:** Prinessa Chellan

**Affiliations:** 1 Department of Chemistry and Polymer Science, Stellenbosch University, Stellenbosch, South Africa

This study attempts to explore the molecular motor, Myosin XXI, using computational methods in order to assist discovery of new therapeutic approaches for leishmaniasis. The authors designed a nanocircuit with a coupled bistable switch inducer, in silico, using Tinker Cell. The basic premise for this study was that the intended targets could be impaired either through antisense RNA for the calmodulin binding motif or protein phosphatase 2A. The simulations carried out showed that the nanocircuit acted as a nanomachine when a chemical gradient was applied. The authors concluded that their designed circuit was found to be a good biological network which could be manipulated to potentially disrupt several processes such as intracellular trafficking, metabolite efflux, motility by Myosin XXI and the survival mechanism of ABC transporters causing mortality of the leishmanial parasite.

 Overall, the article is well written and the experimental approach appears to be well thought out. I cannot comment on the specifics of the computational methods as I have limited experience in this area.

 I recommend indexing of this article as it contributes a new strategy for elimination of the Leishmanial parasite.

I have read this submission. I believe that I have an appropriate level of expertise to confirm that it is of an acceptable scientific standard.

# Peer review: Referee response for version 1

**DOI:** 10.5256/f1000research.11538.r19802

**Published:** 2017-02-15

**Authors:** Jaime J. Seguel

**Affiliations:** 1 Department of Computer Science and Engineering, College of Engineering, University of Puerto Rico at Mayagüez, Mayagüez, Puerto Rico

The article by Kosey and Singh proposes a novel molecular motor intended to impair the activity of Myosin and ABC transporters as agents of Leishmaniasis. A wide array of simulations and measurements seems to support the authors' claims, specifically the claim that the addition of an inducer increases the possibility of avoiding undesired basins of attraction in the circuit's dynamics, and the claim that both, Myosin XXI motility and the metabolite efflux will be hindered as result of the action of the proposed circuit. Design and simulation are by themselves a significant undertaking. Molecular motors such as dynein or myosin are engineered by Nature, and the artificial replication of these remarkable designs is one of the greatest challenges of biological engineering. A still limited understanding of the laws of molecular mechanics at different scales in a biological system prevents a design based on solid theoretical principles. Genetic circuit design is yet an experimental art. And mostly as a result of early experimentation in protein and metabolic engineering, a wide variety of tools that include several computational methods, have been developed and are currently used to predict the effects of a genetic circuit design in a larger biological system. Besides the novelty of the circuit design, this article is an excellent display of the tools and methodological approach to design verification. My sole critique is not about the novelty of the design or the strength of the tools and methodology but rather the limited discussion of the results. I think that the article would be greatly benefited by the addition of interpretations of the statistics and the Boolnet and GRENITS outputs, which in the current version are mostly left to the interpretation of the reader.

I have read this submission. I believe that I have an appropriate level of expertise to confirm that it is of an acceptable scientific standard, however I have significant reservations, as outlined above.
